# Supplementary material for: Twitching motility suppressors reveal a role for FimX in type IV pilus extension dynamics
Source: bioRxiv. 2025 Jul 11:2025.07.10.664058. Preprint. [Version 2] doi: 10.1101/2025.07.10.664058 (PMC12265715; doi:10.1101/2025.07.10.664058)
Supplement: 1 [file NIHPP2025.07.10.664058v2-supplement-1.pdf]

bioRxiv preprint doi: https://doi.org/10.1101/2025.07.10.664058; this version posted July 11, 2025. The copyright holder for this preprint (which was not certified by peer review) is the author/funder, who has granted bioRxiv a license to display the preprint in perpetuity. It is made available under aCC-BY 4.0 International license.

SI Figure Captions

Figure S1: Extension regulatory effector mutants produce no detectable surface pili. Representative SDS-PAGE of sheared surface proteins. Major pilin subunits (PilA) are indicated. Sample loading was normalized to flagellin (FliC) levels.

Figure S2: Mutations which suppress the *fimX* twitching deficit do not confer a growth benefit. Bacterial growth curves across 18 hours in LB media. Points represent the means of triplicate samples from three independent experiments ± SD.

Figure S3: Motility suppressor mutants remain susceptible to pilus-targeting phage PO4. Bacterial growth curves across 18 hours in LB media with PO4 phage-challenge. Points represent the means of triplicate samples from three independent experiments ± SD.

Figure S4: FimX complementation increases twitching motility in original Δ*fimX* suppressor isolates. (A) Quantification of sub-agar stab twitching motility zones for Δ*fimX* twitching suppressor mutants complemented with FimX variants. Representative crystal violet-stained twitching zones are shown to the left. Scale bar = 1 cm. Bars represent the means of triplicate samples from three independent experiments ± SD. **(B)** Representative colonies showing pairwise interactions (pink) between FimX and FimX AAA mutant. PilZ was used as a known non-interacting negative control with FimX (52). Untagged T18/T25 plasmid and PilS-T18/PilS-T25 homodimers (63) were used as negative and positive controls respectively. **EV**: empty pHERD30T vector, ***fimX***: *fimX* in pHERD30T vector, **FimX AAA**: FimX AAA in pHERD30T vector.

Figure S5: Expression of CyaB *in trans* bypasses the Pil-Chp network to increase twitching motility. (A) Quantification of sub-agar stab twitching motility zones for a Δ*cyaB* mutant complemented with CyaB or a catalytically inactive mutant (D234S). Representative CyaB or CyaB D234S-expressing BTH101 colonies are shown in the inset to the right. Pink colour results from increased cAMP levels. Representative crystal violet-stained twitching zones are shown to the left. Bars represent the means of triplicate samples from three independent experiments ± SD. **(B)** Quantification of sub-agar stab twitching motility zones for a Δ*pilGH* double mutant complemented with CyaB. Representative twitching zones are shown to the left. Bars represent the means of triplicate samples from three independent experiments ± SD. All scale bars = 1 cm. *****: $p \leq 0.001$ (Two-tailed parametric *t*-test). **EV**: empty pHERD30T vector, **His_6-CyaB (D234S)**: N-terminally hexa-histidine tagged CyaB or CyaB D234S in pHERD30T vector.

bioRxiv preprint doi: https://doi.org/10.1101/2025.07.10.664058; this version posted July 11, 2025. The copyright holder for this preprint (which was not certified by peer review) is the author/funder, who has granted bioRxiv a license to display the preprint in perpetuity. It is made available under aCC-BY 4.0 International license.

Figure S6: Mutants with an mRuby3 cassette insertion upstream of the *pilMNOPQ* operon remain sensitive to pilus-targeting phage PO4. Bacterial growth curves across 10 hours in LB media with PO4 phage-challenge. Points represent the means of triplicate samples from two independent experiments ± SD. **CyaB R456L**: CyaB R456L in pBADGr vector.

Figure S7: Type II secretion system-dependent clearing on skim milk agar plates for original *fimX* suppressor isolates. (A) Representative colonies and surrounding zones of clearance for the Δ*fimX* twitching suppressor mutants. Scale bar = 1 cm. **(B)** Quantification of the colony area subtracted from the skim milk clearance area. Bars represent the means of duplicate samples from three independent experiments ± SD. *: $0.05 \geq p \geq 0.01$; ***: $0.001 \geq p$ (Two-tailed parametric *t*-test).

Figure S8: Alphafold3-predicted models of PilB supressor mutants *pilB* Δ1bp and PilB T430P. (A) Model of *P. aeruginosa* PilB ($PilB^{Pa}$) monomer. The N1D and linker region (dark grey), N2D (light blue), and CTD (white) are indicated. The *pilB* Δ1bp C-terminal extension is shown in dark blue. PilB T430 side chain is shown in stick and in orange. The model PAE plot is shown to the right. **(B)** *pilB* Δ1bp full length homohexameric Alphafold3 predicted model is shown below with the extended sequence residues highligted in dark blue. The predicted PilC interface is shown on the bottom. The N1Ds and linker regions for each monomer are hidden. The model on the bottom has the two front monomers hidden for clarity. **(C)** X-ray crystal structure of hexameric PilB from *G. metallireducens* (5TSH) with sequence aligned residues of interest N429 ($PilB^{Pa}$ T430) and R430 ($PilB^{Pa}$ R431) highlighted in orange and magenta, respectively. C2-symetric monmers are highlited in the same colours. Dashed lines indicate approximate protomer interfaces.

Figure S9: *pilB* Δ1bp constructs complement twitching motility to at least WT levels. (A) Quantification of sub-agar stab twitching zone areas of a *pilB*::Tn5 mutant overexpressing *pilB* Δ1bp 3′ truncations. Representative crystal violet-stained twitching zones are shown to the left. PilB expression was induced with 0.05% arabinose. Bars represent the means of duplicate samples from three independent experiments ± SD. **(B)** Quantification of sub-agar stab twitching zone areas of WT cells overexpressing *pilB* Δ1bp 3′ truncations. Representative twitching zones are shown to the left. PilB expression was induced with 0.05% arabinose. Bars represent the means of duplicate samples from three independent experiments ± SD. **EV**: empty pHERD30T vector, ***pilB* Δ1bp-3 to 21**: *pilB* Δ1bp-3 to 21 in pHERD30T vector. All scale bars = 1 cm. **ns**: $p \geq 0.05$; **: $0.01 \geq p \geq 0.001$ (Two-tailed parametric *t*-test).

Figure S10: PilB T430X constructs complement twitching motility to at least WT levels. (A) Quantification of sub-agar stab twitching zone areas of a *pilB*::Tn5 mutant overexpressing T430X mutants. Representative crystal violet-stained twitching zones are shown to the left. PilB

bioRxiv preprint doi: https://doi.org/10.1101/2025.07.10.664058; this version posted July 11, 2025. The copyright holder for this preprint (which was not certified by peer review) is the author/funder, who has granted bioRxiv a license to display the preprint in perpetuity. It is made available under aCC-BY 4.0 International license.

expression was induced with 0.05% arabinose. Bars represent the means of duplicate samples from three independent experiments ± SD. **(B)** Quantification of sub-agar stab twitching zone areas of WT cells overexpressing T430X mutants. Representative twitching zones are shown to the left. PilB expression was induced with 0.05% arabinose. Bars represent the means of duplicate samples from three independent experiments ± SD. **PilB T430X**: indicated PilB substitution in pHERD30T vector. All scale bars = 1 cm. **ns**: $p \geq 0.05$; *: $0.05 \geq p \geq 0.01$ (Two-tailed parametric *t*-test).

Figure S11: PilB T429/431X constructs complement twitching motility. (A) Quantification of sub-agar stab twitching zone areas of a *pilB*::Tn5 mutant overexpressing T429/431X mutants. Representative crystal violet-stained twitching zones are shown to the left. PilB expression was induced with 0.05% arabinose. Bars represent the means of duplicate samples from three independent experiments ± SD. **(B)** Quantification of sub-agar stab twitching zone areas of WT cells overexpressing T429/431X mutants. Representative twitching zones are shown to the left. PilB expression was induced with 0.05% arabinose. Bars represent the means of duplicate samples from three independent experiments ± SD. **PilB T429/431X**: indicated PilB substitution in pHERD30T vector. All scale bars = 1 cm. **ns**: $p \geq 0.05$; ***: $0.001 \geq p$ (Two-tailed parametric *t*-test).

Figure S12: Purified ΔN1D-His$_6$-PilB and FimX protein samples. SDS-PAGE of representative protein samples used in the study. Samples are diluted to the same concentrations added to the reaction mixture preparation.

Figure S13: PilA A86C mutants retain twitching motility. Quantification of sub-agar stab twitching zone areas of PilA A86C mutants. Representative crystal violet stained twitching zones are shown to the left. Bars represent the means of triplicate samples from three independent experiments ± SD. Scale bar = 1 cm.

Figure S14: Vector control assays for FimX localization studies. (A) Quantification of sub-agar stab twitching zone areas of Δ*fimX* mutants complemented with N-terminal fusion of mNeonGreen (mNGr) to FimX. Representative crystal violet stained twitching zones are shown to the left. Expression was not induced with arabinose. Bars represent the means of triplicate samples from three independent experiments ± SD. Scale bar = 1 cm. **EV**: empty pHERD30T, **mNGr-FimX (AAA)**: N-terminal fusion of mNeonGreen to FimX or FimX AAA in pHERD30T vector. **(B)** Representative images of Δ*fimX* mutant cells carrying empty pHERD30T with background fluorescence subtracted. Scale bar = 2 µm. ***: $0.001 \geq p$ (Two-tailed parametric *t*-test).

bioRxiv preprint doi: https://doi.org/10.1101/2025.07.10.664058; this version posted July 11, 2025. The copyright holder for this preprint (which was not certified by peer review) is the author/funder, who has granted bioRxiv a license to display the preprint in perpetuity. It is made available under aCC-BY 4.0 International license.

Figure S15: PilB mutations contributing to enhanced twitching motility in Δ*fimX* are rare. **(A)** Total protein sequence length of PilB orthologues in *P. aeruginosa* from the *Pseudomonas*.com database. Sequences which are the same length as PAO1 PilB (566 residues) are indicated in blue. **(B)** Percentage of sequences with an identifiable α-helix-disrupting residue ± three amino acids from the position which aligns with PAO1 PilB T430.
